# CREATE: Enhancing Cognitive Reserve through Assistive Technology in Elderly Care – Introduction to a Primary Prevention Program

**DOI:** 10.1002/alz.092725

**Published:** 2025-01-09

**Authors:** Paolo Taurisano, Chiara Abbatantuono, Daphne Gasparre, Madia Marika Biasi, Giulia Paparella, Vincenzo Dentamaro, Davide Veneto, Donato Impedovo, Maria Fara De Caro

**Affiliations:** ^1^ University of Bari Aldo Moro, Bari Italy; ^2^ University of Bari, Bari Italy

## Abstract

**Background:**

In the international landscape, assistive technologies are often integrated into programs to support elderly individuals with dementia and/or multiple disabilities (Lancioni et al., 2021). However, there is a need to extend these interventions to primary/secondary prevention of such conditions in the elderly population at higher risk. In response to this need, the collaboration between the U.O.S. of Psychology and Clinical Neuropsychology in Bari and the Welfare Association in Levante offers a novel program for active aging based on the use of health tracking smartwatches in the prevention of neurocognitive disorders

**Method:**

We enrolled 10 MCI subjects who were over 65 years old and had them wear a smartwatch for a period of 6 months. The program requires participants to wear smartwatches that track their health using different sensors. These watches monitor various health parameters and provide personalized prompts. The goal of the program is to improve cognitive reserve, promote independence, and increase participants' awareness of their lifestyle. This is all done through a structured intervention and training sessions as part of the C.R.E.A.T.E. program.

**Result:**

The use of affordable and easily accessible everyday technology, such as wristwatches connected to smartphones or PCs, has been proven to create a physical and psychological environment that participants perceive as “protected” and “safe.” This technology provides practical support for daily activities from the beginning of the intervention and helps maintain learned habits over time. Furthermore, the effectiveness of the technology is confirmed by the adoption of systematic indicators and criteria, which verify the observed behavioral changes in participants.

**Conclusion:**

The future integration of wearable technologies, such as smartwatches, with artificial intelligence is becoming increasingly important. This integration allows for interventions to be tailored specifically to each patient, which further enhances the effectiveness of preventive and supportive strategies in promoting active aging.

